# Clinical efficacy and immune response of neoadjuvant camrelizumab plus chemotherapy in resectable locally advanced oesophageal squamous cell carcinoma: a phase 2 trial

**DOI:** 10.1038/s41416-024-02805-5

**Published:** 2024-08-20

**Authors:** Yue-Yun Chen, Pei-Pei Wang, Yang Hu, Yong Yuan, Yu-Shang Yang, Hua-Shan Shi, Qing Hao, Zhen Lin, Jiang-Fang Tian, Yue Zheng, Ting Liu, Pan-Pan Lin, Heng Xu, Xue-Lei Ma, Li Yang, Zhen-Yu Ding

**Affiliations:** 1 Department of Biotherapy, Cancer Center and State Key Laboratory of Biotherapy, West China Hospital, Sichuan University, Chengdu, 610041 Sichuan China; 2 Department of Oncology, Guangzhou First People’s Hospital, School of Biomedical Sciences and Engineering, South China University of Technology, Guangzhou International Campus, Guangzhou, China; 3 Department of Thoracic Surgery, West China Hospital, Sichuan University, Chengdu, China

**Keywords:** Cancer immunotherapy, Oesophageal cancer, Cancer microenvironment

## Abstract

**Background:**

Neoadjuvant immunotherapy is under intensive investigation for esophageal squamous cell carcinoma (ESCC). This study assesses the efficacy and immune response of neoadjuvant immunochemotherapy (nICT) in ESCC.

**Methods:**

In this phase II trial (ChiCTR2100045722), locally advanced ESCC patients receiving nICT were enrolled. The primary endpoint was the pathological complete response (pCR) rate. Multiplexed immunofluorescence, RNA-seq and TCR-seq were conducted to explore the immune response underlying nICT.

**Results:**

Totally 42 patients were enrolled, achieving a 27.0% pCR rate. The 1-year, 2-year DFS and OS rates were 89.2%, 64.4% and 97.3%, 89.2%, respectively. RNA-seq analysis highlighted T-cell activation as the most significantly enriched pathway. The tumour immune microenvironment (TIME) was characterised by high CD4, CD8, Foxp3, and PD-L1 levels, associating with better pathological regression (TRS0/1). TIME was categorised into immune-infiltrating, immune-tolerant, and immune-desert types. Notably, the immune-infiltrating type and tertiary lymphoid structures correlated with improved outcomes. In the context of nICT, TIM-3 negatively influenced treatment efficacy, while elevated TIGIT/PD-1 expression post-nICT correlated positively with CD8+ T cell levels. TCR-seq identified three TCR rearrangements, underscoring the specificity of T-cell responses.

**Conclusions:**

Neoadjuvant camrelizumab plus chemotherapy is effective for locally advanced, resectable ESCC, eliciting profound immune response that closely associated with clinical outcomes.

## Introduction

Oesophageal cancer ranks as the sixth leading cause of cancer-related deaths worldwide. Oesophageal squamous cell carcinoma (ESCC) is the dominant histological subtype, accounting for approximately 90% of all cases in Asia [1]. Neoadjuvant therapy followed by radical surgery is the endorsed treatment for locally advanced ESCC according to international guidelines. However, local recurrence and distant metastasis are common. During the last decade, the efficacy of neoadjuvant chemoradiotherapy (nCRT) was established and recommended as standard of care [2, 3]. However, toxicities and challenges to the subsequent surgery limit its use in clinical settings. Other neoadjuvant treatment modalities are urgently needed.

The extensive application of immune checkpoint inhibitor in clinical practice has revolutionised the therapeutic landscape of many cancers [4–9]. Due to the low incidence of side effects and good patient tolerance, neoadjuvant immunotherapy is attractive and currently extensively studied [10–13]. However, the efficacy, safety and long-term survival of neoadjuvant immunochemotherapy (nICT) for ESCC are still unknown and need to be further clarified.

Tumour immune microenvironment (TIME) is closely related to tumour occurrence, immune escape and therapeutic effect. TIME changes during immunotherapy are a significant clinical and scientific issue that remains unresolved. Neoadjuvant therapy provides a valuable window to dissect the interactions between tumour cells and immune system, especially, tumour-infiltrating lymphocytes (TILs) in TIME.

In this study, we conducted a single-arm, phase II, prospective clinical trial where the efficacy and safety of nICT for ESCC. We further analysed the immune response landscape to explore the changes of TIME behind the chemotherapy combined with camrelizumab in resectable ESCC.

## Materials and methods

### Study design and patients

This was a single-arm, phase II, prospective clinical trial (Clinical trials identifier, ChiCTR2000034311), under the approval from the ethics committee of our hospital. The study was conducted in line with the Declaration of Helsinki. All patients provided written informed consent.

The primary endpoint is the pathological complete response (pCR) rate. Secondary endpoints include overall survival (OS), disease-free survival (DFS), and adverse events (AEs). Additionally, the exploratory endpoint is the immune response to nICT. Patients with locally advanced ESCC were eligible for enrollment. The eligibility criteria of treatment naïve patients were: pathologically confirmed ESCC; ≥18 years old; T3-4NanyM0 or TanyN(+)M0 based on the 8th American joint Committee on cancer (AJCC) tumour-node-metastasis (TNM) classification; Eastern Cooperative Oncology Group (ECOG) performance status (PS) of 0–1; adequate organ function; written informed consent. The exclusion criteria were: the presence of a second primary malignant disease; pregnant women or women preparing for pregnancy; concurrent autoimmune disease or history of chronic autoimmune disease; allergic to study drugs; patients who received corticosteroid (equivalent to prednisone of >10 mg/day) within 14 days prior to the first day of drug administration or patients who are considered as not suitable for participation by the investigators.

### Treatment

Eligible patients received camrelizumab (200 mg, Day 1, Jiangsu Hengrui Pharmaceuticals Co Ltd., China) plus nab-paclitaxel (260 mg/m^2^, Day 1, Hengrui Inc, China) and carboplatin (area under curve of 5 mg/ml/min, D1, Qilu Inc, China) intravenous (IV) every 3 weeks for 2 cycles. All patients received radiographic examinations after 2 cycles of treatment. Surgery was performed 6–8 weeks after the completion of nICT therapy. The decision to surgery was based on the consensus from a multidisciplinary team. In some situations, such as the outbreak of COVID-19 where patients were unable to receive surgery, more cycles of neoadjuvant treatments were allowed. For those considered unsuitable for subsequent surgery, other treatment protocols including definitive radiochemotherapy were suggested by the multidisciplinary team. At 6–8 weeks after completion of nICT, esophagectomy was performed via McKeown or Ivor Lewis depending on the location and extent of the tumour. Standard two-field (abdominal and thoracic) lymph node (LN) resection was performed for all the patients, and cervical LN dissection was highly selected for patients with suspected cervical LN metastasis, assessed by preoperative computed tomography (CT) and ultrasound.

### Outcome measures

Treatment efficacy was monitored through regular thoracic and abdominal enhanced computed tomography examinations. Tumour response was assessed by the treating physician based on Response Evaluation Criteria in Solid Tumors (RECIST 1.1) [14]. Pathologic response after neoadjuvant treatment was evaluated on surgical samples. Two specialised pathologists followed the Becker criteria for tumour regression staging (TRS) [15]. TRS0 indicates complete (0% residual primary tumour, not including lymph nodes); TRS1 indicates subtotal tumour regression (<10% residual tumour per tumour bed); TRS2 indicates partial tumour regression (10–50% residual tumour per tumour bed); TRS3 indicates minimal or no tumour regression (50% residual tumour per tumour bed). Adverse events during the period of perioperative treatment were identified and recorded according to CTC AE 5.0 criteria. Disease-free survival (DFS) was defined as the time from surgery to tumour relapse or death. Overall survival (OS) was defined as the time period from receiving treatment to death.

### Tumour tissue and peripheral blood samples collection

Endoscopic biopsy specimens were obtained before immunochemotherapy and subsequently utilised for RNA-seq. The remaining biopsy specimens and tumour surgical samples, obtained after immunochemotherapy, were formalin fixed and paraffin-embedded for preparation for multiplexed immunofluorescence (mIF). Peripheral blood samples were collected at baseline and after 2 cycles of immunochemotherapy. CPT™ Mononuclear Cell Preparation T Tubes (Thermo Fisher Scientific, San Diego, CA) were used to isolated peripheral blood mononuclear cells (PBMC) according to standard operating procedures for TCR-seq. The liquid nitrogen (tumour specimens) and −80 °C (PBMC) freezers were continuously monitored for temperature.

### RNA-seq analysis

The transcriptome sequencing and corresponding quality control were performed on an Illumina Hiseq X TEN platform (Shanghai Biotechnology Corp., Shanghai, China). The Limma package was used to perform differential expression. The criterion of differential expression: The absolute value of fold-change (FC) > 1.5, *p* < 0.05. ClusterProfiler (v3.6.0) was used for Gene ontology (GO) enrichment analysis of the differentially expressed genes (DEGs). The ESTIMATE was applied to figure out the total score of immune-related cells. Tumour immune infiltration analysis (CIBERSORT [16], R script, java executable, with tool) was used to analysis the difference of 22 types of infiltration immune cells in patients with responsive group and non-responsive group. The results were displayed using the ‘ggplot2’ R package.

### Multiplexed immunofluorescence

Multiplexed immunofluorescence (mIF) was performed on tumour formalin-fixed paraffin-embedded tissue before and after nICT treatment. A commercially available 6-colour fluorescence kit was used following the manufacturer’s protocol (abs50013, Absin Bioscience, China) [17]. All samples were stained for CD8a (D8A8Y, CST), CD4 (EP204, CST), CD20 (E7B7T, CST)/CD68 (D4B9C, CST), Foxp3 (D2W8E, CST), PD-L1 (E1L3N, CST), and DAPI (nuclear counterstain). The co-expression of checkpoint inhibitors was stained in the order as follows: TIM-3 (D5D5R, CST), TIGIT (E5Y1W, CST), PD-L1 (D4W2J, CST), and DAPI (nuclear counterstain). Each sample was scanned at 4x and 20x resolution by a Vectra Polaris imaging platform. InForm2.5.0 software (PerkinElmer/Akoya) was applied to quantitative image analysis of phenotype and score cells based on biomarker expression.

### TCR-seq analysis

TCR sequencing and quality control were conducted by Wuhan Huada Medical Laboratory Co., LTD. Clonotypes were defined as the recognition of complementarity-determining region 3 (CDR3) sequences by unique antigens assembled using specific VJ gene fragments and then constructed from the alignment using software assembly pipelines. Amino acid sequences, nucleotide sequences, counts and frequency of V-J genes, CDR3 length, and Shannon index were analysed. The differential analysis of TCR sequencing used Limma method. The criterion of differential expression: the absolute value of fold-change (FC) > 1.2, *p* < .05. ‘msa’ package was used to sequence alignments of similarity and to analyse the consistency of nucleotide and amino acid sequence of complementarity-determining region 3 (CDR3). ‘ggseqlogo’ package was used to plot diagram of the nucleotide and amino acid sequence.

### Statistical analysis

A sample size of 42 patients was selected to ensure sufficiently narrow confidence intervals (CIs) for clinical outcomes. It was assumed that the pCR rate in this trial might be approximately 25%. Accounting for a dropout rate of up to 15%, enrolling 37 patients would provide 90.2% power (*α* = 0.1) to reject the null hypothesis.

All statistical analyses and graphs were performed using SPSS 25.0 (IBM Inc, Chicago, IL) and R 4.1.1 (https://www.r-project.org/). Kaplan–Meier method was used to construct survival curves, including DFS and OS, and the log-rank test was used for survival analysis. The 95% confidence intervals (95% CI) of rate ratios in two groups calculated by normal approximation method (*n* > 50) or Newcombe–Wilson method (small samples or proportions close to 0 or 1). Double-tailed *p* < 0.05 was considered as statistically significant. Inform 2.5.0 software was used to perform quantitative analysis of mIF. All statistical analyses and graphs were performed using SPSS 25.0 (IBM Inc, Chicago, IL) and R 4.1.1.

## Results

### Patient characteristics

**Fig. 1:**
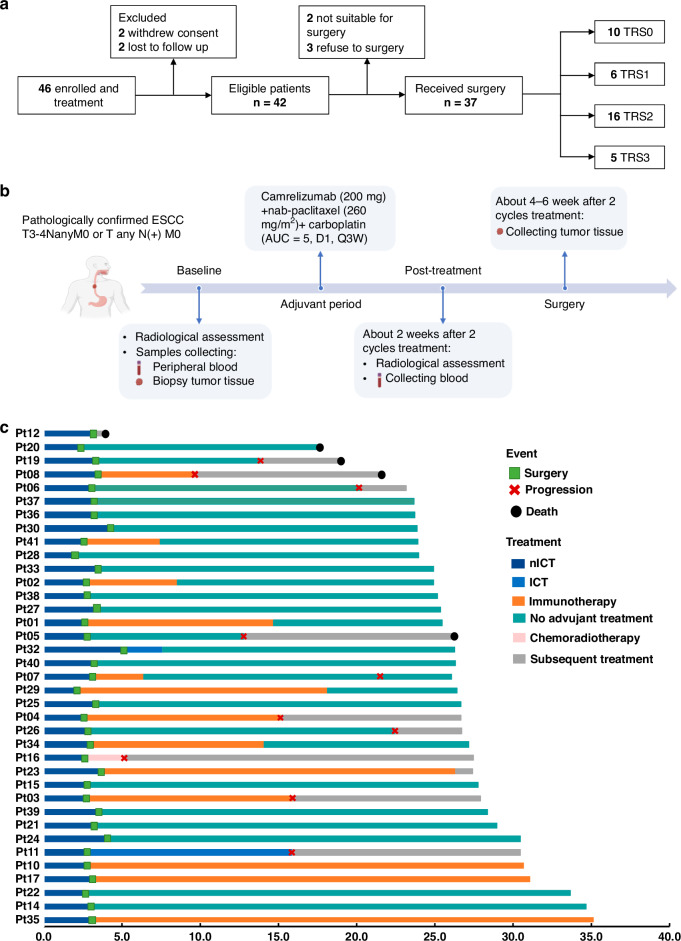
The study design. **a** Flowchart for screening of eligible patients. TRS tumour regression grade. TRS0 indicates complete regression; TRS1 indicates near-complete regression; TRS2 indicates partial regression; TRS3 indicates negligible or no regression; **b** Trial schema. Eligible patients were treated with 2 cycle of neoadjuvant therapy of camrelizumab (200 mg, Day 1) plus nab-paclitaxel (260 mg/m^2^, Day 1) and carboplatin (AUC = 5 mg/mL/min, Day 1), followed by surgical resection. Radiological assessment was performed at the time of baseline, and 2 weeks after 2 cycles therapy and before surgery. Tumour samples were collected at baseline and at the time of surgery. Peripheral blood samples were collected at baseline and 2 weeks after 2 cycles therapy; ESCC oesophageal squamous cell carcinoma, AUC area under the curve. **c** Treatment regimen in the neoadjuvant and adjuvant settings, and follow-up status per patient (*n* = 37); nICT neoadjuvant immunochemotherapy, ICT immunochemotherapy.

Forty-six patients were enrolled and received treatment from Jan 2021 to Jan 2022. Of these patients, 42 were eligible for inclusion in the study and provided written informed consent. Finally, 37 patients who were accomplished the 2 cycles neoadjuvant therapy of camrelizumab (200 mg, Day 1) combined with nab-paclitaxel (260 mg/m^2^, Day 1) and carboplatin (area under curve of 5 mg/mL/min, Day 1) followed by surgery, were enrolled for the analysis in this study (Fig. 1a, b). The detailed treatment process of adjuvant therapy and follow-up status of each patient were summarised in a swimmer’s plot (Fig. 1c). In this cohort, the median age was 66 years (range, 47–79 years). The majority of patients were male, representing 83.8%, and a significant portion had a history of smoking or alcohol consumption, each accounting for 59.5%. A subset of the population, 13.5%, was diagnosed with T4a oesophageal lesions, while a substantial 81.1% presented with nodal involvement N(+). Furthermore, 35.1% of the patients were classified with stage IV disease according to the 8th edition of the AJCC criteria. The lesions were predominantly located in the middle and lower oesophagus, comprising 89.2% of cases (Supplementary Table S1).

### Clinical efficacy

**Fig. 2:**
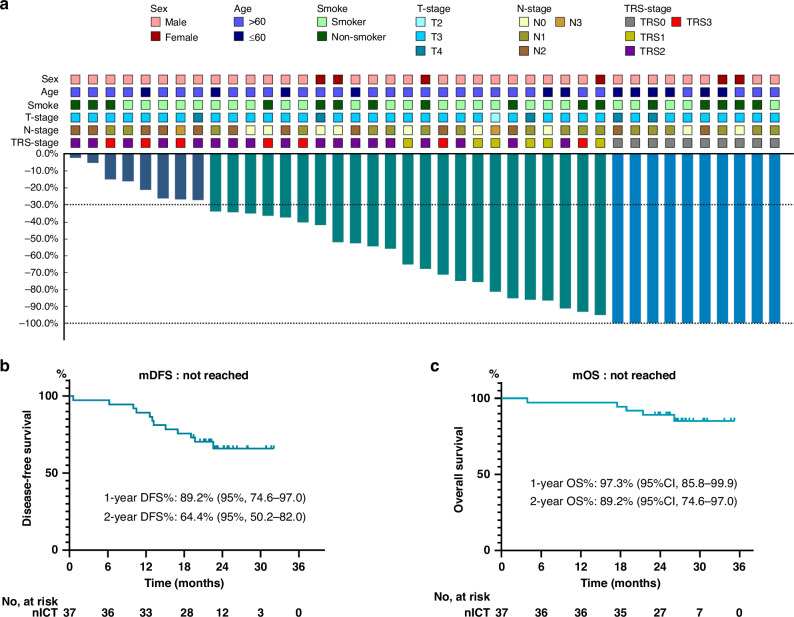
Clinical efficacy. **a** Waterfall plot of radiographic tumour regression (*n* = 37). Each bar represents one patient. The upper column shows clinical characteristics; TRS tumour regression grade, CR complete response, PR partial response, SD stable disease. **b** Disease-free survival for the surgical population (*n* = 37). **c** Overall survival for the surgical population (*n* = 37). mDFS median disease-free survival, mOS median overall survival.

Radiographic assessment revealed that 27.0% of patients achieved complete response (CR) and 51.4% achieved partial response (PR), while stable disease (SD) was observed in 21.6%. There were no instances of disease progression during the neoadjuvant phase. The disease control rate (DCR) stood at 100%, with the objective response rate (ORR) reaching 78.4% as depicted in Fig. 2a.

Of the cohort, 88.1% underwent surgical procedures, with 97.3% attaining R0 resection status. A pCR rate was achieved by 27.0% of patient post-surgery, as further detailed in the primary tumour pathological responses and clinical characteristics illustrated in Fig. 2a. Non-surgical choices were due to patient refusal (*n* = 3), tumour location (*n* = 1), and inadequate pulmonary function (*n* = 1). Definite radiochemotherapy was prescribed to the latter 2 patients.

As of the data cutoff on October 15, 2023, and with a median follow-up period of 26.7 months (range, 23.2–35.2 months), the 1-year and 2-year DFS rates stood at 89.2% (95% CI, 74.6–97.0) and 64.4% (95% CI, 50.2–82.0), respectively, as shown in Fig. 2b, with OS rates at 97.3% (95% CI, 85.8–99.9) and 89.2% (95% CI, 74.6–97.0), correspondingly displayed in Fig. 2c. The median OS and DFS have not yet been reached.

We further analysed the pCR rate in responder and non-responder groups based on clinical efficacy evaluation (CR/PR vs. SD/PD). The pCR rates in the responder and non-responder groups were 32.2% and 0.0%, respectively. The overall AE rates in the responder and non-responder groups were similar (87.1% vs. 83.4%, *p* = 0.495). However, Grades 3–4 AEs were more common in the responder group (32.3% vs. 16.7%, *p* = 0.018). The responder groups showed an obvious tendency towards prolonged DFS and OS compared to the non-responder group (Supplementary Fig. 1).

### Safety

Treatment-related adverse events (TRAEs) of any grade occurred in 86.5% of patients (32 out of 37), with the majority (62.5%) experiencing only mild to moderate side effects (grades 1–2). The most severe AEs (grades 3–4) were cases of myelosuppression, chiefly leukopenia and neutropenia, which accounted for 27.0% of such events. Other common TRAEs included musculoskeletal pain (*n* = 8) and decreased appetite (*n* = 8), increased cholesterol and fatigue (*n* = 5), rash (*n* = 4), and alopecia (*n* = 4), all of which were generally mild and manageable. The AEs most frequently associated with camrelizumab were instances of reactive cutaneous capillary endothelial proliferation (RCCEP), occurring in 17 patients, with the vast majority (94.1%) being classified as grade 1–2. One patient died of severe postoperative pneumonia, while the other patients who experienced severe AEs recovered following appropriate therapy (Supplementary Table S2). Notably, no surgical procedures were delayed due to TRAEs.

### T cell activation in RNA-seq

**Fig. 3:**
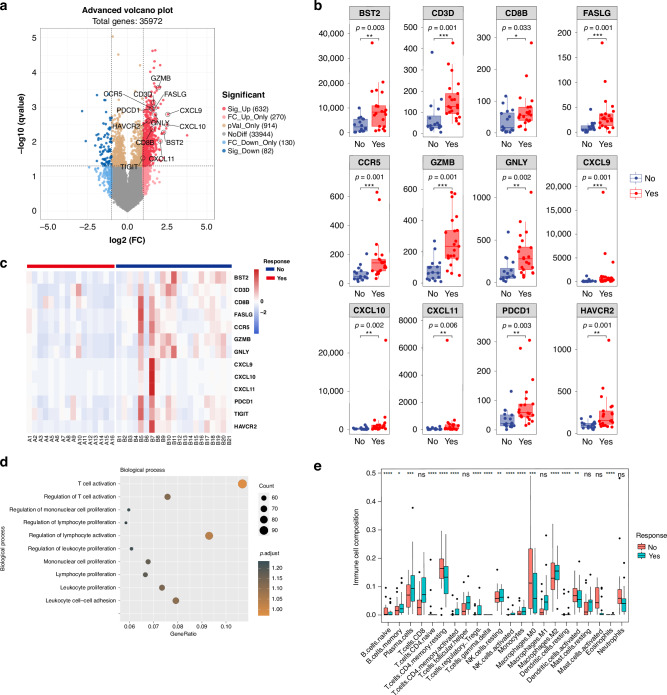
RNA-seq analysis of tumour specimens at baseline (*n* = 37). **a** Differential gene expression between responders (CR/PR, *n* = 21) and non-responder group (SD, *n* = 16); The upregulated immune-related genes were marked. A cut-off of gene expression fold change of ≥1.5 and a false discovery rate (FDR) *q* < 0.05 was applied to select DEGs; **b** Typical immune-related genes in radiographic responders and non-responders; **c** Up-regulation of immune-related genes in patients with radiographic responders and non-responders; **d** The GO enrichment of RNA-seq showing the top 10 up-regulation immune-related genes in the section of biological process; **e** 22 types of infiltration immune cells in responders and non-responders. Responders were those having complete or partial response by RECIST 1.1, while non-responders having stable disease or disease. *p < 0.05, **p < 0.01, ***p < 0.001, asterisks (*) stand for significance levels. The statistical difference of two groups was compared through the Wilcox test.

RNA-seq was performed on tumour samples before nICT treatment (*n* = 37, out of the 46 patients). According to radiographic responses, patients were divided into responder (CR/PR, *n* = 21) or non-responder group (SD, *n* = 16). Eleven immune-related genes were differentially expressed between the two groups, involving T-cytotoxic cells (CD3D, CD8D), T-cell chemokines (CXCL9, CXCL10, CXCL11 and CCR5), T cell activation degranulation (GZMB, GNLY), T cell killing related factors (FASLG) and B cell proliferation (BST2) (Fig. 3a–c). Among these genes, T cell activation and lymphocyte activation regulation pathways were the most prominently enriched (Fig. 3d and Supplementary Fig. 2). In the responder group, the expression profile of the activated CD4^+^ T cells and memory B cells was elevated, while that of the resting memory CD4^+^ T cells decreased. Additionally, we observed a discernible trend of augmentation in CD8+ T cells and M1 macrophages within the responder group (Fig. 3e).

### T cell infiltration and TIME subtypes

**Fig. 4:**
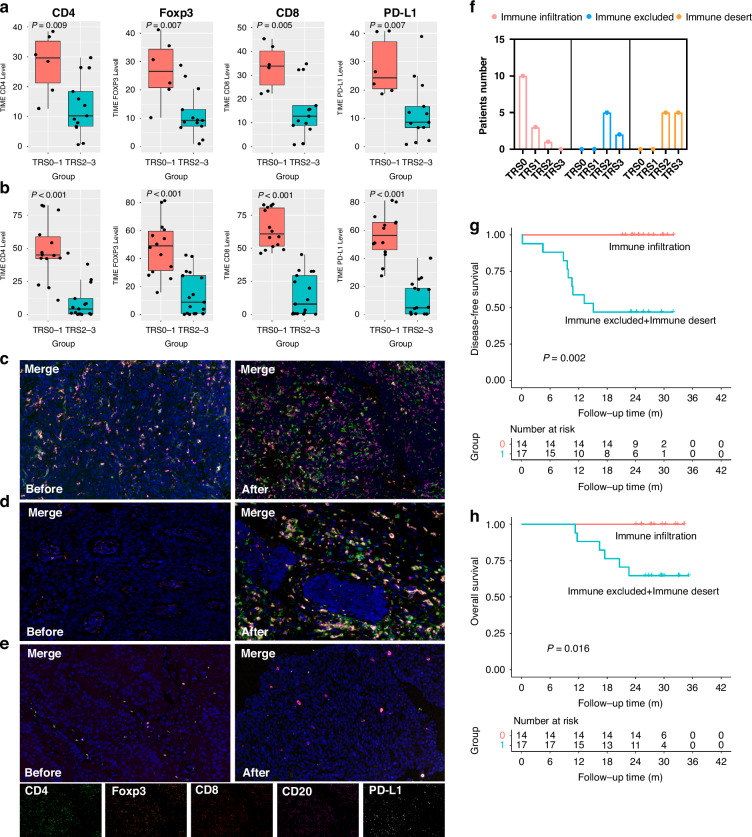
T cell infiltration and TIME types in the tumour tissue by mIF. Expression of immune cell markers in TIME: CD4, Foxp3, CD8 and PD-L1 expression in ESCC tumour samples before (**a**, *n* = 19) and after (**b**, *n* = 31) nICT treatment; three TIME types based on the infiltration of immune cells in the tumour tissue before and after immunochemotherapy by mIF: immune infiltration (**c**, *n* = 14); immune excluded (**d**, *n* = 7); immune desert (**e**, *n* = 10)； **f** TIME type grouped by TRS (*n* = 31); **g** Disease-free survival in different TIME types (*n* = 31); **h** Overall survival in different TIME types (*n* = 31): mIF multiplexed immunofluorescence, TIME tumour immune microenvironment, TRS tumour regression stage. CD4 (green), Foxp3 (orange), CD8 (red), CD20 (rose-red) and PD-L1 (white).

The expression of CD4, CD8, Foxp3 and PD-L1 was strongly associated with the pathological response, both in the pre- and post-treatment TIME (Fig. 4a, b). High expression of CD20 and CD68 in the pre-treatment tumour samples was positively associated with the pathological response (Supplementary Fig. 3).

Here, based on the infiltration of immune cells, including T cells (CD4/CD8), Treg cells (Foxp3), B cells (CD20) and macrophages (CD68), adopting criteria from a previous report [18] with minor modification, we classified the TIME into three types: immune infiltrating (remarkable surge in the infiltration of immune cells and CD8+ T cell in postoperative tumour tissue or scarred area of residual tumour, Fig. 4c), immune tolerance (infiltration of immune cells are detectable surrounding the tumour tissue, not in tumour tissue, Fig. 4d) and immune desert (rare infiltration of immune cells and CD8+ T cell markers before and after treatment, Fig. 4e). Ten patients (71.5%) in the immune-infiltrating type had pathological complete response (TRS0), 3 patients (21.4%) had major pathological response (TRS1), and 1 patient (7.1%) had TRS2. For the other TIME types, most patients had TRS2 or TRS3 (Fig. 4f). In addition, patients of immune infiltration type had an obvious DFS (Fig. 4g, *p* = 0.002) and OS (Fig. 4h, *p* = 0.016) advantage over those in the other TIME type.

### Tertiary lymphoid structures (TLSs)

**Fig. 5:**
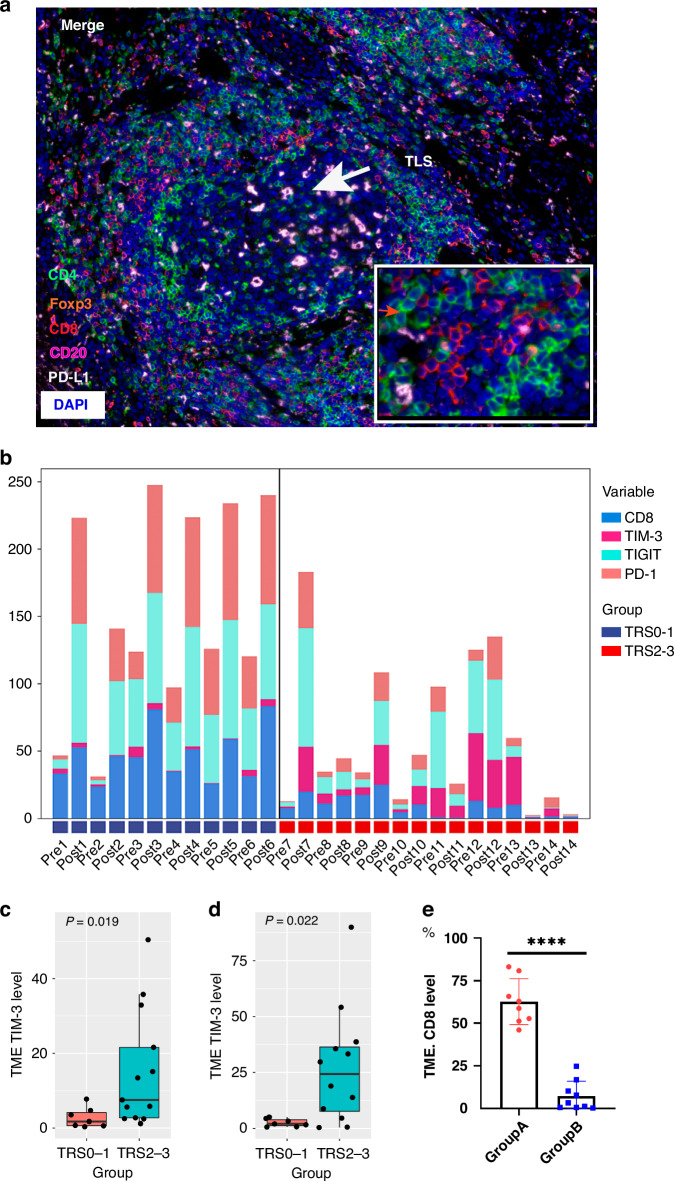
TLSs, TIM-3/TIGIT/PD-1 expression and TRS in tumour specimen before and after nICT. **a** A presentative figure of TLSs in TIME in nICT group. TLSs tertiary lymphoid structures; CD4 (green), Foxp3 (orange), CD8 (red), CD20 (rose-red) and PD-L1 (white). **b** Expression of TIM-3/TIGIT/PD-1 before and after nICT treatment in a good (TRS0) and a poor (TRS3) pathological responder; compared to those with stable TIGIT/PD-1 expression (*n* = 9) after nICT treatment, patients with significant elevated TIGIT/PD-1 expression (*n* = 8) had higher expression of CD8 in tumour specimen (****p < 0.001); increased expression of TIM-3 was associated with poor efficacy before (**c**, *n* = 20) or after (**d**, *n* = 19) nICT treatment; **e** After nICT treatment, patients with significantly elevated TIGIT/PD-1 expression (Group A, *n* = 8) had higher CD8 expression in tumour specimens compared to who showed no significant change in TIGIT/PD-1 expression (Group B, *n* = 9); nICT neoadjuvant immunochemotherapy, TIME tumour immune microenvironment.

TLSs refer to immune cell aggregates within non-lymphoid tissue structures, which can facilitate the influx of immune cells into tumour sites [19]. Therefore, TLSs within or adjacent to tumour tissues are considered closely associated with the immunotherapeutic efficacy against tumours. In post-surgical tumour specimens, TLSs were easily identified in the patients with pathological response of TRS0/1 (*n* = 14, Fig. 5a), characterised by T and B cells infiltration.

### TIM-3/TIGIT/PD-1 immune checkpoint expression

Previously, we showed that the co-expression of TIM-3/PD-L1 and TIGIT/PD-L1 was negatively related to the prognosis of ESCC patients [20]. In this study, high expression of both TIGIT and PD-1, along with low expression level of TIM-3 after treatment indicated better efficacy (TRS0) of nICT. However, simultaneous high expression of TIGIT, PD-1, and TIM-3 after treatment resulted in TRS3 (Supplementary Fig. 4 and Fig. 5b). High levels of TIM-3 both pre- and post-treatment were often accompanied by a low level of CD8+ T cells expression, resulting in an unfavourable efficacy (TRS2/3) (Fig. 5c, d). Patients with significant elevated TIGIT and PD-1 expression (*p* < 0.05) after treatment also had higher expression level of CD8 (Fig. 5e).

### Specific T cell clones

**Fig. 6:**
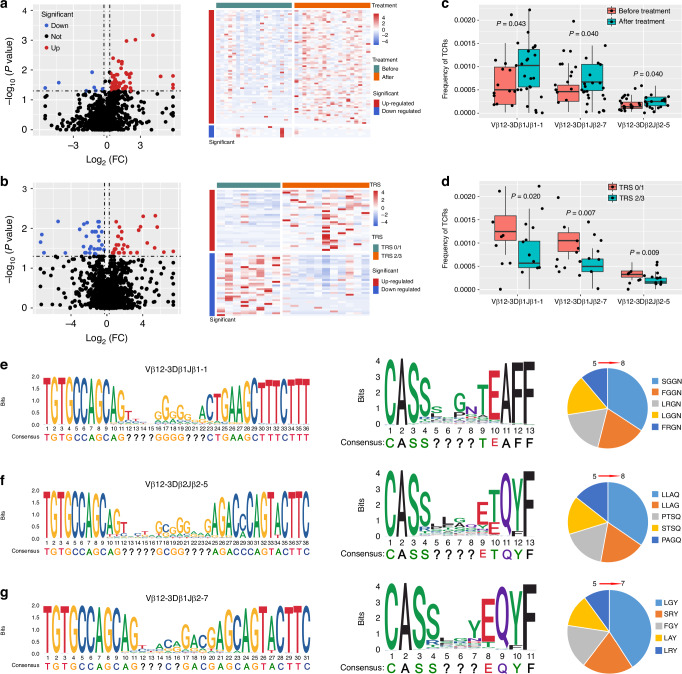
TCR-seq analysis of 19 paired peripheral blood samples before and after nICT treatment. **a** Differential expression analysis of TCR clonality before and after nICT treatment; **b** Differential expression analysis of TCR clonality between pathological responders (TRS0/1) and non-responders (TRS2/3). **c** The frequency of these shared TCRs was increased after treatment (*n* = 38); **d** Higher frequency of three shared TCRs in patients with pathological response (TRS0/1) (*n* = 19); sequence motif analysis of high-frequency groups of TCRs Local alignment was applied to calculate the similarity of the base (left) and amino acid (middle) of Vβ12-3Dβ1Jβ1-1 (**e**), Vβ12-3Dβ1Jβ2-7 (**f**) and Vβ12-3Dβ2Jβ2-5 (**g**). The question marks indicated that the sequence at that position is not conservative. The percentage of top 5 non-conserved amino acid sequences (right) in patients with high frequency of TCRs was shown.

T cells play a pivotal role in tumour immunotherapy. Complementarity-determining region 3 (CDR3) is a major determinant of TCR diversity, and an important determinant of antigen recognition. We subsequently conducted TCR-seq on 20 paired peripheral blood samples collected before and after nICT treatment (1 patient was excluded due to no surgery). Regarding TCR diversity, nICT treatment led to an increase in Shannon index in all samples, independent of pathological response (TRS0/1 or TRS2/3, Supplementary Fig. 5). TCR clonality has the potential to predict immunotherapeutic effect. In this study, less TCR clones were observed in the pathological responders (TRS0/1) and the number of unique clones increased after nICT treatment (Supplementary Fig. 6). We found that 72 TCR clones were upregulated and 7 down-regulated after nICT treatment. Compared with non-responders (TRS2/3), 33 TCR clones were upregulated and 34 down-regulated in pathological responders (TRS0/1, Fig. 6a, b). High frequencies of Vβ12-3Dβ1Jβ1-1, Vβ12-3Dβ1Jβ2-7 and Vβ12-3Dβ2Jβ2-5 were significantly associated with pathological regression (TRS0/1) and these TCR rearrangements increased after nICT treatment (Fig. 6c, d). To determine clonotype contributions and further identify the non-conserved sequence of these 3 TCR rearrangements, we detailed the nucleotides, amino acid sequence and percentages of the top 5 non-conserved amino acid sequences of the 3 shared TCRs (Fig. 6e–g).

## Discussion

In this study, we conducted a prospective clinical trial to investigated the clinical efficacy and immune response of camrelizumab combined with chemotherapy in patients with resectable ESCC. Of the 42 enrolled, 37 successfully underwent surgical resection, achieving an R0 resection rate of 97.3%. Among those who received radical surgery, a pCR was achieved in 10 (27.0%) patients. Treatment-related adverse events of grade 3–4 were reported in 29.7% of cases, predominantly manifesting as leukopenia and neutropenia, which were amenable to treatment. Immune-related adverse events were generally mild and manageable. Survival analysis revealed 1-year and 2-year disease-free survival (DFS) and overall survival (OS) rates of 89.2%, 64.4%, and 97.3%, 89.2%, respectively, underscoring the therapeutic potential of the regimen. Subsequent RNA-sequencing analysis illuminated the T-cell activation pathway as the preeminent enriched process. To delve deeper into the role of T-cells in immunotherapy efficacy, we discerned that the infiltration of lymphocytes within the tumour immune microenvironment (TIME) was pivotal. This was particularly true for those with established tertiary lymphoid structures (TLSs), which garnered the most substantial immunotherapeutic benefit. Beyond the mere presence of lymphocytes, the modulation of their function emerged as equally vital. The expression of immune checkpoints, together with the activation of T cells, played a significant role in dictating therapeutic outcomes. Finally, we attempted to determine the T cell clones underneath the immunochemotherapy, and we identified three specific clones as the most likely candidates. Our data suggest that specific T-cell subtypes, especially those situated in TIME, are critical to the success of neoadjuvant immunotherapy.

Neoadjuvant immunotherapy for ESCC was under intensive study. The first study immunotherapy combined with canonical nCRT reported a pCR of 46.1% in 28 patients [21]. A similar pCR of 55.6% was reported in another PALACE-1 study of neoadjuvant immunotherapy combined with chemoradiotherapy [22]. These pCR rates were comparable to that in CROSS study, where immunotherapy was not used [2]. In addition, toxicities were of concern. In the 1st study, 2 patients died of pulmonary toxicities. Alternately, due to the favourable safety profile, nICT became the mainstay neoadjuvant therapy for locally advanced ESCC in clinical studies. Some exploratory clinical studies were reported successively. In these studies, the pCR rate ranged from 16.0 to 45.0%, with low overall AEs [10–13, 23, 24]. Recently, an exploratory study (NATION-1907) was reported where solo PD-L1 inhibitor was used in the neoadjuvant setting [25]. This elegant study showed excellent tolerance and significantly prolonged survival, with a 92% 2-year OS rate in a highly selected ESCC patient cohort (*n* = 25). However, long-term survival was rarely reported in these studies. In our study, the pCR rate was 27.0%, consistent with pCR rates from similar studies [10–13, 23, 24]. Neoadjuvant immunochemotherapy resulted in improved pCR rates over neoadjuvant chemotherapy (nCT), though not as high as those observed with nCRT. The phase III JCOG 1109 trial underscored a critical observation: the nCRT group exhibited significantly higher pCR rates compared to the nCT group, yet there was no discernible difference in overall survival [26]. In this regard, the long-term survival of neoadjuvant immunochemotherapy warranted further elucidation, especially compared with the survival of nCT or nCRT.

In the phase III Checkmate 577 study, adjuvant nivolumab significantly improved DFS in patients with pathological positive tumour or nodal disease after nCRT [27]. It would be interesting to ask if immunotherapy would be applied as a neoadjuvant or adjuvant therapy. The data were lacking to compare the difference between these 2 situations. It was argued that immunotherapy would be appropriate when tumour neoantigens were present and TIME remained intact. Thus, the long-term efficacy of neoadjuvant immunotherapy was urgently waited, would be helpful for the decision of pre- or postoperative immunotherapy.

Not just the presence but also the functional status of the infiltrated lymphocytes was crucial. In a clinical trial of melanoma, increased expression of chemokines CXCL9 and CXCL10 after neoadjuvant immunotherapy was highly associated with antitumor efficacy [28]. These findings were consistent with ours. In our study, extensive immune-related genes especially those related to T cells killing (CD3D, CD8D, FASLG) and regulation-related (CXCL9, CXCL10, CXCL11, GZMB, GNLY) were significant upregulated in responders (CR/PR) compared to non-responders (SD/PD). In support of this, the highly-regulated genes were prominently enriched in the pathways of T cell activation and regulation.

There has been no in-depth study of the composition of TIME during the nICT in ESCC. We revealed that the high expression of CD4 and CD8 in TIME before treatment was closely related to the pathological response. In addition, TILs represented by CD4^+^ and CD8^+^ T cells infiltration were significantly increased in some patients after nICT. These patients were more likely to achieve pathological response. nICT not only stimulated TIL infiltration, but also induced the dynamic changes in the spatial structure of TIME. The TIME was further classified into three types: immune infiltrating, immune tolerance or immune desert, based on the infiltration of immune cells after nICT. These TIME types were reported in other tumour types before [18, 25, 29], but never described for ESCC before our study. Our study also found that patients with immune tolerance or immune desert TIME types had poorer prognosis compared to those with immune infiltrating types. However, effective postoperative treatments for patients with poor response to nICT remain unknown. Previous research has indicated that combining anti-angiogenic agents [30] or cancer vaccines [31] may offer therapeutic benefits for these patients. Building on this foundation, we conducted a prospective clinical study to assess the efficacy of neoantigen dendritic cell (DC) vaccines as adjuvant therapy for ESCC.

In patients with melanoma and sarcoma, TLSs formation after immunotherapy was strongly related to the antitumor efficacy. T cells and B cells infiltration, as well as with the spatial organisation and compartment, all contributed to the final immune response [32–34]. In our study, TLSs were closely associated with pathological response (TRS0/1) in nICT therapy. We had reasons to suspect that the TLSs had a unique role in modulating the anti-tumour immune response profiles, favoring the durable anti-tumour effects observed in the nICT group. Building on the results observed in this study, we will conduct further research to delineate the specific mechanisms which tertiary lymphoid structures affect the efficacy of immunotherapy.

Persistent exposure to tumour antigen induced expression of immune checkpoints and effector T-cell exhaustion [35, 36]. TIM-3 was considered to be an important biomarker of T-cell exhaustion. The elevated expression of TIM-3 inhibited the activation of CD8^+^ T cells [37, 38]. Here, we found that high expression of TIM-3 both before and after neoadjuvant immunotherapy was accompanied by low expression of CD8 T cells, also with an unfavourable prognosis. Both TIGIT and PD-1 are critical immune checkpoints, playing an important role in the regulation of T cells [39–41]. In this study, we found that significant elevated TIGIT/PD-1 expression after nICT treatment was usually accompanied by higher expression of CD8 T cells, which also response well to nICT. The local regulatory T-cell feedback circuit was considered playing a vital role in immune homoeostasis [42].

Number of TCR clones are strongly positive linked with immunotherapy efficacy [43–45]. Shared TCR sequences between tumour and peripheral blood were found correlated with favourite survival outcomes [46]. In a clinical trial exploring neoadjuvant immunotherapy for lung cancer, a positive correlation between the proportion of shared TCR clonotypes and pathological response was found [47]. In our study, unique TCR clonality increased after nICT treatment, including 3 shared TCR clonotypes. These shared TCR clonotypes were significantly associated with pathological regression. Our results, together with others, demonstrated that expanded TCR clonalities were closely associated with immunotherapy efficacy. In particular, the increased shared TCR clones were considered to be specific T cell clones that were responsible for the efficacy. We believed these clones would be very helpful for adjuvant therapy. Currently, we are conducting an adjuvant therapy trial in ESCC by analysing the specificity of the shared TCR clones.

Our study has some limitations. Firstly, it was designed as a single-arm trial which lacked a control group. Secondly, our sample size was relatively small. In addition, because of the short follow-up period, the long-term survival benefit of neoadjuvant immunochemotherapy remains unclear.

In conclusion, we study showed that neoadjuvant camrelizumab combined with chemotherapy was an effective treatment for locally advanced ESCC patients with acceptable and manageable adverse events. In addition, it’s elicited profound immune response that closely associated with clinical efficacy, which might be a potential biomarker in neoadjuvant immunotherapy setting.

## Supplementary information

Supplementary materials

## Data Availability

The data underlying this article are available in the article and in its online Supplementary Material. The code referenced in the article has been uploaded to GitHub (https://github.com/haoqing12/TRB_analiysis.git).
