# Supplementary material for: Clinical efficacy and immune response of neoadjuvant camrelizumab plus chemotherapy in resectable locally advanced oesophageal squamous cell carcinoma: a phase 2 trial
Source: Br J Cancer. 2024 Aug 20;131(7):1126–36. doi: 10.1038/s41416-024-02805-5 (PMC11442672; doi:10.1038/s41416-024-02805-5)
Supplement: Supplementary file 1 — Supplementary materials [file 41416_2024_2805_MOESM1_ESM.pdf]

Supplementary materials

A

Pathohistological Results	Responder N=31	Non-responder N=6
**Margin status**	n (%)	
**R0**	31(100.0)	5(83.3)
**Histological response**		
**TRS0**	10(32.2)	0(0.0)
**TRS1**	6(19.4)	0(0.0)
**TRS2**	11(35.5)	5(83.3)
**TRS3**	4(12.9)	1(16.7)
**MPR**	16(51.6)	0(0.0)

MPR: major pathological remission

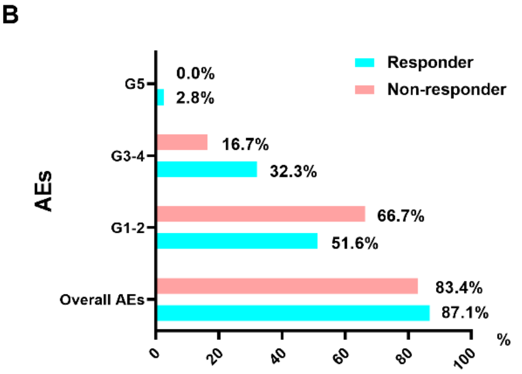

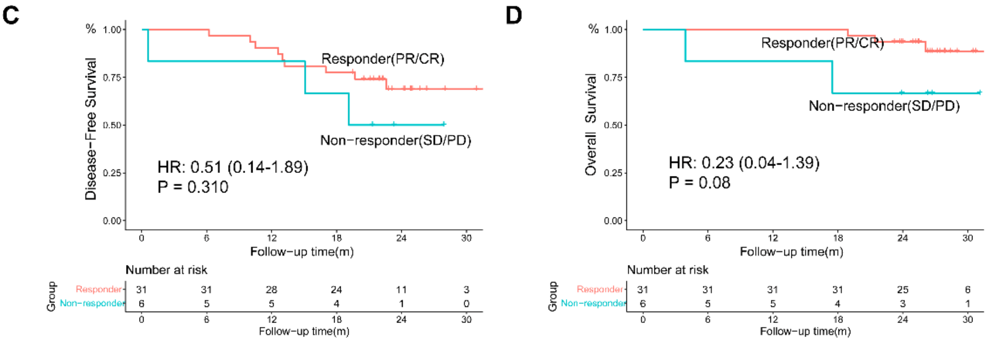

Supplementary Figure 1. Postoperative pathological grading, AE incidence rate and survival analysis of responder and non-responder groups according to clinical efficacy evaluation (CR/PR vs. SD/PD). (A) Postoperative pathological grading; (B) Adverse Event (AE) incidence rate; (C) Disease-free survival (DFS); (D) Overall survival (OS); HR, hazard ratio.

A

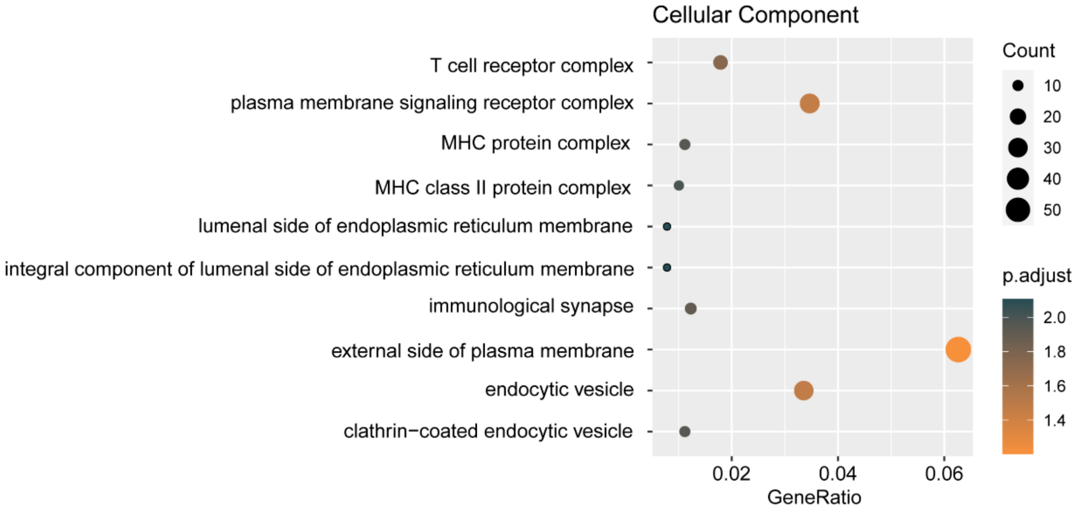

B

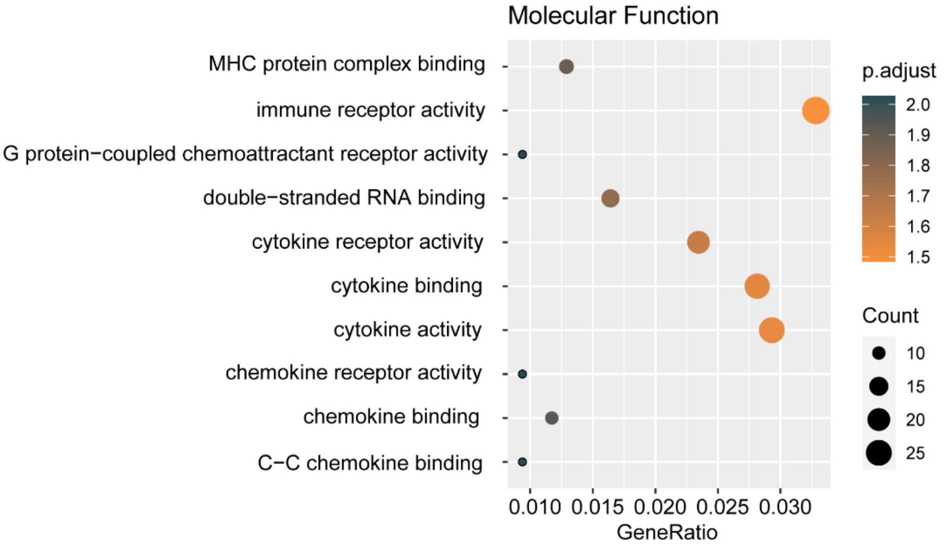

Supplementary Figure 2. Enrichment of pathways for the top 10 immune-related genes

The top 10 up-regulated immune-related genes in the sections of cellular component (A) and molecular function (B).

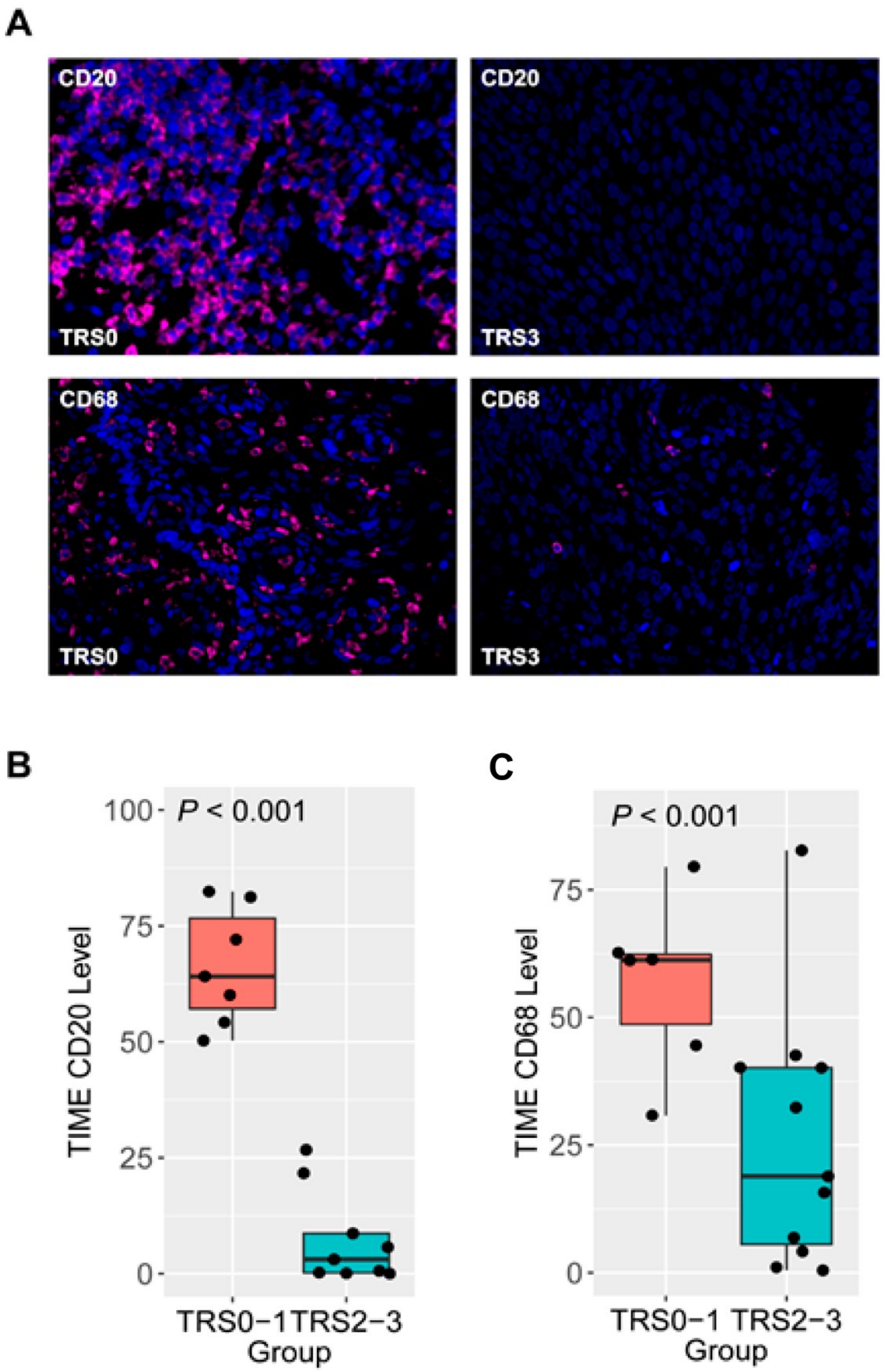

Supplementary Figure 3. The distribution CD20 or CD68 expression and pathological responses

(A) The typical presentation of CD20 or CD68 expression in patients with TRS0 or TRS3; Either CD20 (B, n=16) or CD68 (C, n=17) expression was associated the pathological responses; TIME, tumor immune microenvironment; TRS, tumor regression stage.

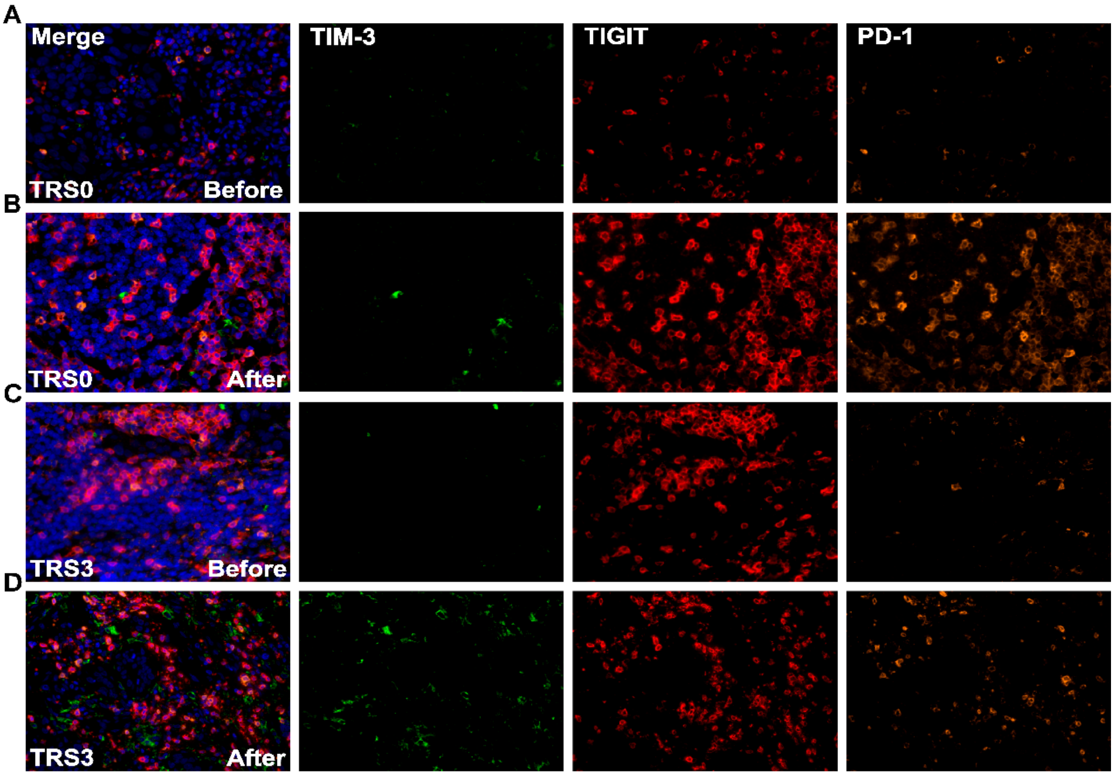

Supplementary Figure 4. Expression of TIM-3/TIGIT/PD-1 before and after nICT treatment in a good (TRS0) and a poor (TRS3) pathological responder;

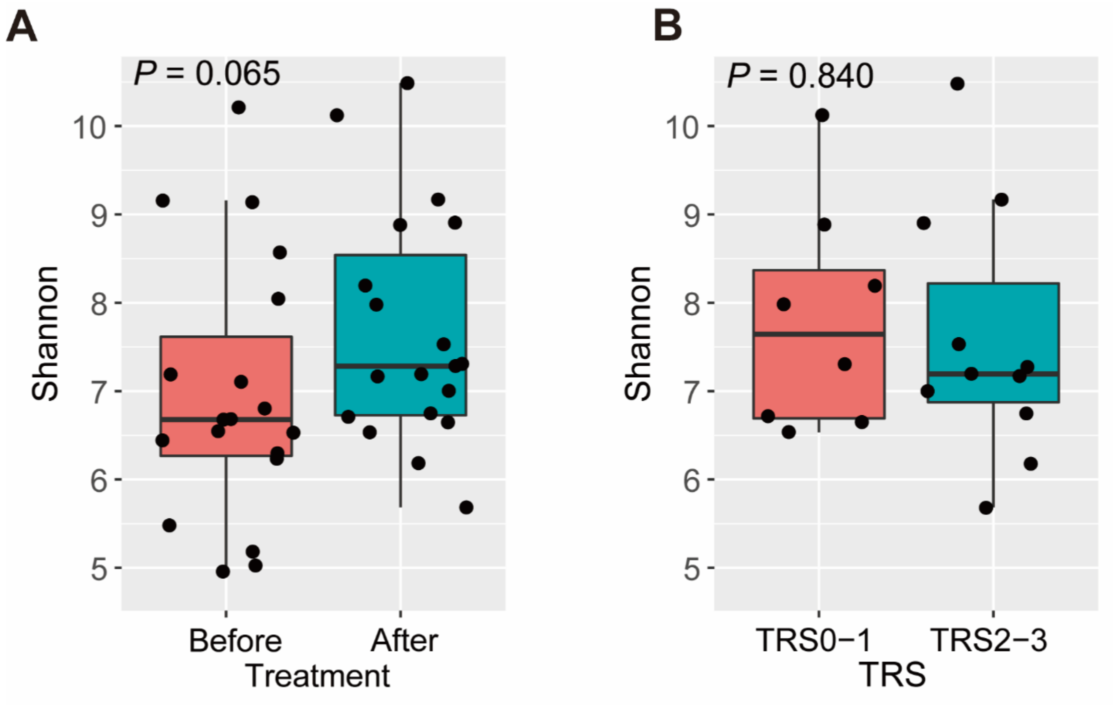

Supplementary Figure 5. The diversity of CDR3 sequence

(A) Shannon index increased after nICT (n=38); (B) Shannon index was stable between pathological responders (TRS0/1) and non-responders (TRS2/3, n=19). CDR3, complementarity determining region 3.

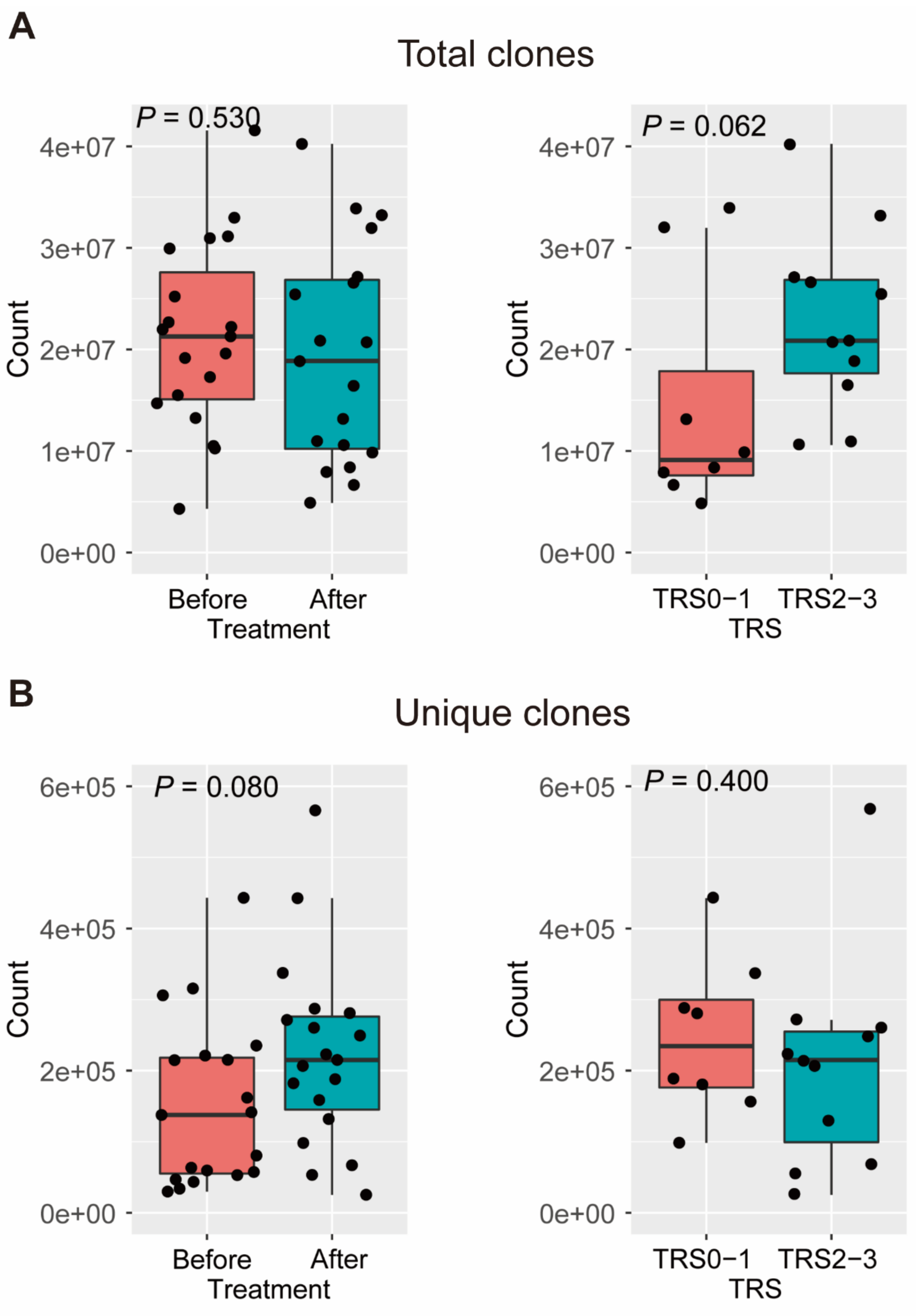

Supplementary Figure 6. TCR clones before and after nICT

TCR clones were assayed on 19 paired samples before and after nICT. (A) Total clones showed no difference (n=38), while fewer were detected in pathological responders (TRS0/1); (B) The number of unique clones increased after nICT (n=38), but no obvious difference was observed between pathological responders (TRS0/1) and non-responders (TRS2/3, n=19); CDR3, complementarity determining region3; **TRS, tumor regression stage;** nICT,neoadjuvant immunochemotherapy.

Table S1. Demographic characteristics of the population

Characteristics	n (%)
**Age**	
≤ 65 years	17 (45.9)
>65 years	20 (54.1)
**Sex**	
Male	31 (83.8)
Female	6 (16.2)
**Smoking**	
No	15 (40.5)
Yes	22 (59.5)
**Alcohol**	
No	15 (40.5)
Yes	22 (59.5)
**Primary site**	
Upper	4 (10.8)
Middle	19 (51.4)
Lower	14 (37.8)
**BMI**	
≤18.5	8 (21.6)
>18.5	29 (78.4)
**TNM Stage**	
II-III	24 (64.9)
IV	13 (35.1)
**T stage**	
T2-3	32 (86.5)
T4	5 (13.5)
**N stage**	
N0	7 (18.9)
N1-3	30 (81.1)

BMI, body mass index; TNM indicates tumor node metastasis.

Table S2: Adverse events of the population

Adverse Events n (%)	G1-2	G3-4	G5
**All AEs**	20 (54.1)	11 (29.7)	1 (2.7) *
**Led to discontinuation**	0 (0.0)	0 (0.0)	0 (0.0)
**Led to death (postoperative)**	0 (0.0)	0 (0.0)	1 (2.7) *
**Chemotherapy-related AEs**			
Myelosuppression	4 (10.8)	10 (27.0)	0 (0.0)
Musculoskeletal pain	8 (21.6)	0 (0.0)	0 (0.0)
Decreased appetite	8 (21.6)	0 (0.0)	0 (0.0)
CHOL increased	5 (13.5)	0 (0.0)	0 (0.0)
Rash	4 (10.8)	0 (0.0)	0 (0.0)
Alopecia	4 (10.8)	0 (0.0)	0 (0.0)
Fatigue	3 (8.1)	0 (0.0)	0 (0.0)
Nausea	3 (8.1)	0 (0.0)	0 (0.0)
Numbness	2 (5.4)	0 (0.0)	0 (0.0)
Pruritus	2 (5.4)	0 (0.0)	0 (0.0)
Increased BIL	2 (5.4)	0 (0.0)	0 (0.0)
ALT increased	1 (2.7)	0 (0.0)	0 (0.0)
Noninfectious Fever	1 (2.7)	0 (0.0)	0 (0.0)
**Immune-medicated AEs**			
REECP	16 (43.2)	1 (2.7)	0 (0.0)
Pneumonitis	1 (2.7)	1 (2.7)	0 (0.0)
Thyroid dysfunction	2 (5.4)	0 (0.0)	0 (0.0)

*One patient died of severe pneumonia after surgery. Abbreviation: AEs, adverse events; CHOL: Cholesterol; BIL: Bilirubin; ALT: Alanine aminotransferase; ALT, alanine transaminase; REECP: reactive cutaneous capillary endothelial proliferation.
